# Response: Commentary: Viewing photos and reading nouns of natural graspable objects similarly modulate motor responses

**DOI:** 10.3389/fnhum.2015.00524

**Published:** 2015-09-24

**Authors:** Giovanni Buccino, Barbara F. M. Marino

**Affiliations:** ^1^Dipartimento di Scienze Mediche e Chirurgiche, Università “Magna Graecia” di CatanzaroCatanzaro, Italy; ^2^IRCCS NeuromedPozzilli, Italy; ^3^Dipartimento di Psicologia, University BicoccaMilano, Italy

**Keywords:** nouns, affordance, objects, embodiment and grounded cognition, semantics

In two behavioral experiments, Marino et al. (2014) investigated the modulation of the motor cortex during the semantic processing of graspable and non-graspable objects, presented either as photos or written nouns. They used scrambled images and pseudowords as control stimuli. At 150 ms after stimulus presentation, participants had to respond when the stimulus referred to a real object with their right (experiment 1) or left (experiment 2) index finger, and refrain from responding when a scrambled image or a pseudoword was presented. Participants' responses related to photos or nouns of graspable objects were slower than those related to non-graspable ones, independent of the responding hand. According to the authors, these findings support the notion that the semantic processing of photos and written nouns referring to graspable objects, is due to common neural substrates, crucially involving the motor system. Specifically, they forward that, to solve the requested semantic task, participants relied on the motor representations of potential hand interactions with the object depicted in the photo (affordance as described by Gibson, 1977) or expressed by the verbal label. In this way, the motor system was engaged in two tasks, that is processing stimuli and performing a motor response (pressing the button), at the same time. Participants therefore paid a cost as revealed by the slowing down of their motor responses.

Some previous papers (Tucker and Ellis, 2004; Makris et al., 2011) support the notion that the recruitment of the motor system (affordance effect) during the visual presentation of objects appears later than 150 ms after stimulus presentation. Based on that, in his commentary Makris (2015) proposes an alternative explanation of the experimental findings reported by Marino et al. (2014), namely as due to an attentional effect. The author proposes that graspable objects suddenly appearing on screen can automatically grab exogenous attention (Yantis and Jonides, 1984) and then for a rapid period after stimulus onset (~100–150 ms) a withdrawal of attention from the objects in display occurs, leading to a rebalance of the affordance-driven motor plans. According to Makris, it could be that 150 ms after the presentation of the stimuli, exogenous-like attention was withdrawn only from the graspable, but not the non-graspable, objects. This way, participants would have to re-direct their attention to the graspable objects in order to resolve the semantic task and this process would have some cost in the timing of their responses. According to the author, this account would fit with a theoretical model known as the affordance competition hypothesis proposed by Cisek (2007).

In principle, one cannot rule out the attentional hypothesis, as an alternative explanation to the findings of Marino et al. (2014). However, even admitting a specific role of attention in explaining the data, in the commentary by Makris it is not clear why, at difference with non-graspable objects, the processing of graspable ones would require the withdrawal of attention at about 150 ms after stimulus presentation and the subsequent reallocation of attention to solve the semantic task. It is worth keeping in mind that also non-graspable objects were presented abruptly and therefore they could potentially grab exogenous attention as graspable objects did. Moreover, the affordance competition hypothesis does not seem to support this time course of attention allocation since in this account action selection and specification are parallel and not serial processes. That said, it is worth stressing as Makris himself admits, that it is difficult to disentangle between attentional and motor processes. Based on several studies, one may argue that there is no need to postulate two control mechanisms, one for action and one for attention. Rather, attention derives from the activity of the sensorimotor circuits devoted to interact with objects (e.g., Corbetta et al., 1998; Craighero et al., 1999).

The time course of the recruitment of the motor system during semantic processing of objects and nouns is still a matter of debate. However, there is increasing evidence of an early involvement of the motor system during semantic tasks involving language material. Neurophysiological studies (for review see Pulvermüeller et al., 2009) support a recruitment of the motor system during language processing of words related to action within the first 200 ms after stimulus onset. In the same time window, behavioral studies have shown that participants give slower hand motor responses when they have to process language material expressing hand actions or hand related objects (as for nouns, see Marino et al., 2013). In a very recent paper (Klepp et al., 2015), by means of magnetoencephalography, it has been assessed that this early slowing down of motor responses is due to a suppression of beta rhythm weaker than that found during the preparation and execution of actual movements. Taken together, these findings may lead to the conclusion that the modulation of the motor system during language processing may change over time, moving from an early interference, operating between 100 and 200 ms after stimulus onset, to a subsequently facilitation, operating later than 200 ms after stimulus presentation (Chersi et al., 2010). As for seen objects, the recruitment of the motor system (affordance effect), has been clearly shown at 200 ms after stimulus presentation (Buccino et al., 2009), that is quite earlier than 300 ms found by Makris et al. (2011). In addition, the findings of Marino et al. (2014) strongly suggest that there is a specific modulation of motor responses during the processing of photos depicting graspable objects already 150 ms after stimulus presentation. This modulation parallels the one occurring during the processing of language material expressing the same object category. Hence the proposal that the neural substrates devoted to processing photos depicting graspable objects and nouns referring to the same object category may be shared and crucially involve the motor system. Future studies should assess at what extent the semantic processing of seen objects and nouns overlap.

## Conflict of interest statement

The authors declare that the research was conducted in the absence of any commercial or financial relationships that could be construed as a potential conflict of interest.
